# Toll-like receptor 4-dependent upregulation of cytokines in a transgenic mouse model of Alzheimer's disease

**DOI:** 10.1186/1742-2094-5-23

**Published:** 2008-05-29

**Authors:** Jing-Ji Jin, Hong-Duck Kim, J Adam Maxwell, Ling Li, Ken-ichiro Fukuchi

**Affiliations:** 1 Department of Cancer Biology and Pharmacology, University of Illinois College of Medicine at Peoria, Box 1649, Peoria, IL 61656, USA; 2 Department of Medicine, School of Medicine, University of Alabama at Birmingham, Birmingham, Alabama 35294-0024, USA

## Abstract

**Background:**

Aβ deposits in the brains of patients with Alzheimer's disease (AD) are closely associated with innate immune responses such as activated microglia and increased cytokines. Accumulating evidence supports the hypothesis that innate immune/inflammatory responses play a pivotal role in the pathogenesis of AD: either beneficial or harmful effects on the AD progression. The molecular mechanisms by which the innate immune system modulates the AD progression are not well understood. Toll-like receptors (TLRs) are first-line molecules for initiating the innate immune responses. When activated through TLR signaling, microglia respond to pathogens and damaged host cells by secreting chemokines and cytokines and express the co-stimulatory molecules needed for protective immune responses to pathogens and efficient clearance of damaged tissues. We previously demonstrated that an AD mouse model homozygous for a destructive mutation of TLR4 has increases in diffuse and fibrillar Aβ deposits as well as buffer-soluble and insoluble Aβ in the brain as compared with a TLR4 wild-type AD mouse model. Here, we investigated the roles of TLR4 in Aβ-induced upregulation of cytokines and chemokines, Aβ-induced activation of microglia and astrocytes and Aβ-induced immigration of leukocytes.

**Methods:**

Using the same model, levels of cytokines and chemokines in the brain were determined by multiplex cytokine/chemokine array. Activation of microglia and astrocytes and immigration of leukocytes were determined by immunoblotting and immunohistochemistry followed by densitometry and morphometry, respectively.

**Results:**

Levels of tumor necrosis factor (TNF)-α, interleukin (IL)-1β, IL-10 and IL-17 in the brains of TLR4 wild-type AD mice were significantly higher than those in TLR4 wild-type non-transgenic littermates. Such increases in cytokines were not found in TLR4 mutant AD mice as compared with TLR4 mutant non-transgenic littermates. Although expression levels of CD11b (a microglia marker) and GFAP (a reactive astrocyte marker) in the brains of TLR4 mutant AD mice were higher than those in TLR4 wild type AD mice, no difference was found in levels of CD45 (common leukocyte antigen).

**Conclusion:**

This is the first demonstration of TLR4-dependent upregulation of cytokines in an AD mouse model. Our results suggest that TLR4 signaling is involved in AD progression and that TLR4 signaling can be a new therapeutic target for AD.

## Background

Patients with Alzheimer's disease (AD) develop deposits of aggregated amyloid β-protein (Aβ) in neuritic plaques and cerebral vessels (cerebrovascular amyloid angiopathy). Fibrillar Aβ deposits in AD brain are accompanied by innate immune responses such as activated microglia and increased levels of cytokines [1]. Earlier studies indicated that the deposition of Aβ in the brain might activate microglia, initiating a proinflammatory cascade that resulted in the release of potentially cytotoxic molecules, cytokines, complements, proteases and other acute phase proteins, ultimately causing neurodegeneration [1,2]. In accordance with this view, long-term use of nonsteroidal anti-inflammatory drugs (NSAIDs) reduced the risk of AD and delayed its onset [3-8]. Overexpression of mutant forms of amyloid β-protein precursor (APP) in the brains of transgenic mice produced amyloid plaques surrounded by activated microglia and reactive astrocytes and upregulated interleukin (IL)-1, IL-6 and tumor necrosis factor (TNF)-α, which resembled the alterations found in patients with AD [9]. When Tg2576 mice, an AD mouse model, overexpressing a mutant form of APP were treated with an NSAID, ibuprofen, the mice improved in open field activity and had reductions in levels of IL-1β, reactive astrocytes and Aβ load [10,11]. The deletion of the TNF type 1 death receptor gene in APP23 transgenic mice, another transgenic mouse model of AD, inhibited Aβ generation, decreased amyloid plaques and prevented learning and memory deficits [12]. The deletion of the interferon (IFN)-γ receptor type I gene in Tg2576 mice caused less gliosis and amyloid plaques. In IFN-γ receptor wild-type Tg2576 mice, IFN-γ elicited TNF-α secretion resulting in upregulation of β-site APP-cleaving enzyme (BACE1) in astrocytes, which caused an increase in Aβ production. Additionally, upregulation of IFN-γ and TNF-α suppressed Aβ degradation by microglia in the latter mice [13]. These observations support the notion that upregulation of proinflammatory cytokines and activation of glial cells promote the disease progression.

Recently, however, the reports of the potential beneficial effects of innate immune responses in AD are increasing. Activation of cultured microglia with toll-like receptor 2 (TLR2), TLR4, or TLR9 ligand markedly boosted ingestion of Aβ [14-17]. An acute injection of lipopolysaccharide (LPS, a TLR4 ligand) in the hippocampus reduced Aβ load in an AD transgenic model [18] and microglial activation is required for the LPS-induced reduction of Aβ load [19]. Accumulation of Aβ in the brain of an AD mouse model triggered chemoattraction of bone marrow-derived cells (microglia) that restricted amyloid deposits in the brain [20]. Upon activation with LPS, bone marrow-derived microglia decreased Aβ load in the brain of an AD mouse model [21]. Mononuclear cells from normal subjects were able to clear Aβ from the sections of AD brain but those from AD patients were not. Furthermore, Aβ upregulates expression of β-1,4-mannosyl-glycoprotein 4-β-N-acetylglucosaminyltransferase (MGAT3) and TLRs in mononuclear cells from normal subjects, whereas these genes were down-regulated in cells from AD patients [22], suggesting immune defects in AD patients. The deletion of the CCR2 [a receptor for monocyte chemoattractant protein-1 (MCP-1 or CCL2)] gene in Tg2576 mice reduced the number of microglia and impaired Aβ clearance in the brain [23]. Overexpression of C3 inhibitory soluble complement receptor-related protein inhibited C3 in the brain of an AD mouse model, decreased microglial activation and increased Aβ deposition and neurodegeneration [24]. Overproduction of transforming growth factor (TGF)-β1 in an AD mouse model resulted in a vigorous microglial activation that was accompanied by at least a 50% reduction in Aβ load [25]. Overexpression of IL-1β in the hippocampus of APPswe PS1dE9 mouse model of AD [26] activated microglia and astrocytes, upregulated expression of TNF-β and IL-6 and decreased amyloid plaques [27]. These reports suggest that neuroinflammation found in AD brain is beneficial and that activation of microglia and astrocytes as well as upregulation of cytokines and chemokines can be therapeutic.

TLRs function as pattern-recognition receptors in the innate immune system [28]. Activated phagocytes and tissue dendritic cells through TLR signaling respond to pathogens and damaged host cells by secreting chemokines and cytokines and express the co-stimulatory molecules needed for protective immune responses, efficient clearance of damaged tissues and adaptive immunity [29-31]. Microglia is the phagocyte in the central nervous system and activated by a number of TLR ligands [32,33]. We previously demonstrated that an AD mouse model (Mo/Hu APPswe PS1dE9 mice) homozygous for a destructive mutation of TLR4 (*Tlr*^*Lps*-*d*^/*Tlr*^*Lps*-*d*^) had increases in diffuse and fibrillar Aβ deposits as well as buffer-soluble and insoluble Aβ in the brain as compared with a TLR4 wild-type AD model mice [17]. Because induction of TLR signaling activates microglia and upregulates cytokines and chemokines, in the present study, we investigated the roles of TLR4 in activation of microglia and astrocytes as well as upregulation of cytokines and chemokines in the AD mouse model. Activation of microglia and astrocytes was determined by immunohistochemistry and immunoblotting. Levels of cytokines and chemokines were quantified by multi-plex cytokine array.

## Methods

### Animals

A pathogen-free transgenic line of AD mouse model, Mo/Hu APPswe PS1dE9 mice [26], was obtained from Jackson Laboratory (Bar Harbor, ME) and maintained by crossing transgenic males with B6C3F1 females that were purchased from Jackson Lab, also. C3H/HeJ mice are highly susceptible to Gram-negative infection and resistant to bacterial lipopolysaccharide (LPS) due to a destructive mutation of the TLR4 gene (*TlrLps-d*). The genotyping for the APPswe transgene was performed by the PCR-based method provided by the Jackson Lab. The TLR4 genotype was determined by PCR followed by restriction enzyme digestion with Nla III as described previously [17]. The transgenic mice express chimeric mouse/human APP with the double mutations (K670N and M671L) and human PS1 with a deletion of exon 9 found in familial AD patients. In this study, four experimental groups of 13–15 month-old mice which differed in the TLR4 gene and Mo/Hu APPswe PS1dE9 transgene were used. The four experimental groups were (1) homozygous TLR4 mutant Mo/Hu APPswe PS1dE9 transgenic mice (n = 8), designated here as TLR4m AD mice for simplicity, (2) TLR4 wild-type transgenic mice (n = 10), designated as TLR4w AD mice, (3) homozygous TLR4 mutant non-transgenic littermates (n = 9), designated as TLR4m non-AD mice and (4) TLR4 wild-type non-transgenic mice (n = 9), designated as TLR4w non-AD mice.

### Brain tissue lysate preparation

Mice were sacrificed by lethal injection of pentobarbital and the brains were quickly removed. After removing the cerebellums, the left hemispheres were processed for protein array and immunoblot analyses. The left cerebrum was lysed using the Bio-Plex cell lysis kit (Bio-Rad, Hercules, CA). Briefly, the cerebrum was rinsed with the cell wash buffer and cut into 3 × 3 mm pieces. The sample was transferred to a Dounce homogenizer on ice and homogenized in the lysis buffer containing proteinase inhibiters. Then the ground tissue sample was transferred to a clean microcentrifuge tube and frozen at -80°C. After thawing, the sample was sonicated on ice for 30 seconds. The sample was centrifuged at 4,500 g for 10 minutes at 4°C, and the supernatant was collected for the protein array and immunoblotting.

### Immunoblotting and densitometric analysis

Immunoblot analysis was used to quantify cerebral CD11b, CD45 and glial fibrillary acidic protein (GFAP) in AD mice. Protein concentrations of brain lysates were determined by Bio-Rad Protein Assay (Bio-Rad). Five micrograms of total protein from each sample were applied to 10–20% Tris-HCl gradient SDS-PAGE and electrotransferred to polyvinylidine difluoride (PVDF) membranes (Millipore, Bedford, MA). The membranes were blocked by phosphate buffered saline (PBS) containing 5% non-fat dried milk (w/v), 0.02% sodium azide, and 0.02% Tween 20 for 1 hr at room temperature, incubated at 4°C overnight with primary antibodies CD11b (Serotec, MCA711, Raleigh, NC) for detection of activated microglia, CD45 (Serotec, MCA1031G) for detection of migratory leukocytes or GFAP (Dako, Demark) for reactive astrocytes and visualized by the western lighting chemiluminescence reagent plus (Perkin Elmer, Boston, MA) according to the manufacturer's protocol. The membranes were reprobed with monoclonal antibody against glyceraldehyde-3-phosphate dehydrogenase (GAPDH) (Chemicon, Temecula, CA). The optical densities of CD11b, CD45, GFAP and GAPDH bands from the membranes were determined by densitometric scanning using a HP Scanjet G3010 Photo Scanner and HP Photosmart Software. The optical density of each protein band was divided by that of the GAPDH band on the same lane from the same membrane for normalization. The ratios were expressed as mean ± standard error. Intergroup differences were assessed by analysis of variance (ANOVA) and two-tailed Student's t-test. P < 0.05 was considered statistically significant.

### Cytokine/chemokine microarray

Cerebral tissue lysates were prepared and their protein concentrations were determined as described above. Levels of cytokines and chemokines in the cerebral tissue lysates were determined by the bead-based suspension microarray technology (AssayGate, Ijamsville, MD) [34]. The threshold of detection was determined by adding two standard deviations to the mean fluorescence intensity of twenty zero standard (background) replicates. The minimum detectable concentrations were 3.6 pg/ml for IL-1α, 3.8 pg/ml for IL-1β, 2.3 pg/ml for IL-2, 2.5 pg/ml for IL-3, 4.3 pg/ml for IL-4, 1.3 pg/ml for IL-6, 8.2 pg/ml for IL-10, 11 pg/ml for IL-12 (p40), 3.5 pg/ml for IL-12(p70), 9.2 pg/ml for IL-17, 6.6 pg/ml for granulocyte macrophage colony-stimulating factor (GM-CSF), 2.3 pg/ml for IFN-γ, 16 pg/ml for TGF-β1, 3.7 pg/ml for TNF-α, 2.5 pg/ml for MCP-1, 1.3 pg/ml for macrophage inflammatory protein (MIP)-1α and 3.1 pg/ml MIP-1β. The results were expressed in picograms per milligram of brain protein and analyzed by ANOVA and two-tailed Student's t-test using SigmaStat for Windows Version 3.00. P < 0.05 was considered statistically significant.

### Immunohistochemistry

Frozen sections (5 μm thickness) were prepared and subjected to the immunoperoxidase method. Endogenous peroxidase was eliminated by treatment with 3% H_2_O_2_/10% methanol Tris-buffered saline (TBS) for 20 min at room temperature. After washing with water and 0.1 M TBS (pH 7.4), slides were blocked with 2% bovine serum albumin (BSA) and 2% goat serum in 0.1% triton-X-100 TBS (TBST) buffer for 60 min at room temperature to prevent non specific protein binding. Then the slides were incubated with primary antibody CD11b (1:200), CD45 (1: 1000), or GFAP (1:1000) in 2% BSA, 2% goat serum TBST overnight at 4°C. The sections were rinsed in 0.1 M TBST containing 0.1% BSA and incubated with biotinylated secondary antibody anti-rat IgG (1:200) for CD11b, anti-rat IgG (1:1500) for CD45 or anti-rabbit IgG (1:1000) for GFAP in 2% BSA, 1% goat serum TBST for 1 h at room temperature. Finally, the avidin biotin peroxidase method using 3,3'-diaminobenzidine as a substrate (Vector, Burlingame, CA) was performed according to manufacturer's protocol. For the negative control, slides were processed without primary antibody.

Histomorphometry for quantification of activated microglia and reactive astrocytes was performed using an OLYMPUS IX71 microscope, OLYMPUS DP70 digital camera and the Image Pro Plus v4 image analysis software (Media Cybernetics, Silver Spring, MD) capable of color segmentation and automation via programmable macros. Four to five coronal brain sections, each separated by more than 240 μm interval, from each mouse were analyzed. All brain sections contained both cerebral cortex and hippocampus. Consecutive 4 pictures of the cerebral cortex with no overlaps, starting from the midline were taken from each brain section using a 10× objective and 1× eyepiece lens. Pictures in which folded tissues were found were discarded. Thus, approximately 15 pictures of the cerebral cortex from each mouse were analyzed. The area of the cerebral cortex in the pictures varied from approximately 0.5 mm^2 ^to 1 mm^2^. CD11b, CD45 and GFAP stained areas were expressed as a percentage of total cerebral cortex examined. Data were expressed as mean ± standard error as a bar graph. Intergroup differences were assessed by ANOVA and two-tailed Student's t-test. P < 0.05 was considered statistically significant.

## Results

### Activated microglia

**Figure 1:**
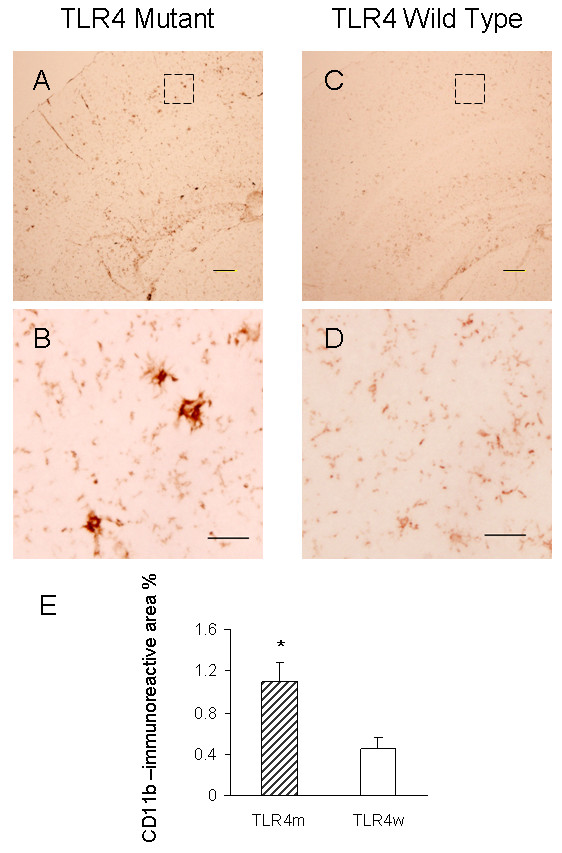
CD11b immunoreactive microglia in TLR4m and TLR4w AD mice. The frozen sections of cerebral cortices from TLR4m (A and B) and TLR4w (C and D) AD mice were stained with anti-CD11b antibody. Scale bars are 50 μm in A and C, and 10 μm in B and D. The middle images (B and D) are a high magnification of the areas indicated by the squares in the top images (A and C). CD11b immunoreactive area percentages of TLR4m and TLR4w AD mice were shown as a bar graph (means ± SE, * P < 0.05) (E). Approximately 15 fields of the cerebral cortex (0.5 to 1 mm^2 ^each, using a 10× objective and 1× eyepiece lens) from 4 – 5 coronal brain sections, each separated by more than 240 μm interval, from each mouse were analyzed. TLR4m and TLR4w represent TLR4m and TLR4w AD mice, respectively.

**Figure 2:**
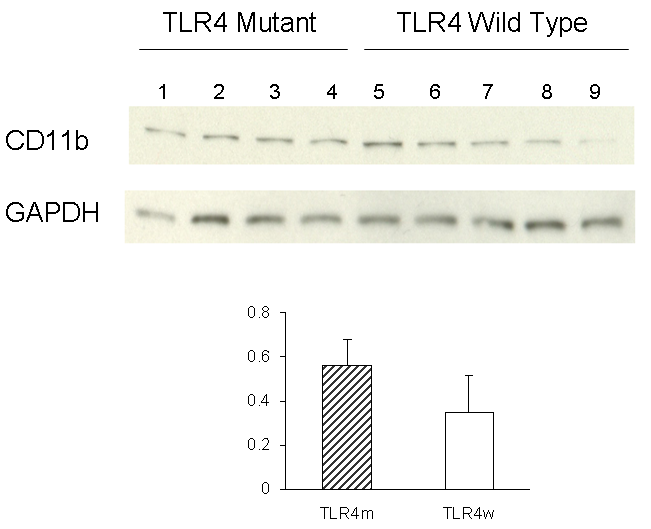
Levels of CD11b and GAPDH in the cerebral tissue lysates were determined by immunoblotting using anti-CD11b and anti-GAPDH antibodies, respectively. The bar graph represents densitometric quantification of CD11b after normalization with GAPDH (means ± SE). The mean of CD11b levels in TLR4m AD mice is greater than that in TLR4w AD mice but not statistically significant. Lane 1 through 4 are tissue lysates from TLR4m AD mice and lane 5 through 9 are from TLR4w AD mice.

Activated microglia were analyzed for expression of CD11b (Mac-1) by immunohistochemistry. Representative micrographs from the cerebral cortex of AD mice were shown in Fig. 1 A through D. Morphometric analysis of the CD11b immunostaining revealed that the cerebral cortex of TLR4m AD mice (1.10 ± 0.18%) had greater microglial immunoreactivity than TLR4w AD mice (0.45 ± 0.10%, P = 0.021) (Fig. 1E). CD11b immunoreactivity in the brains of TLR4m and TLR4w non-AD mice was unremarkable (data not shown). Microglial activation was further determined by immunoblot analysis (Fig. 2). Expression levels of CD11b in the cerebral tissue lysates from TLR4m AD mice were on average higher than those from TLR4w AD mice, which were not statistically significant due to large variances (Fig. 2).

### Migratory leukocytes

**Figure 3:**
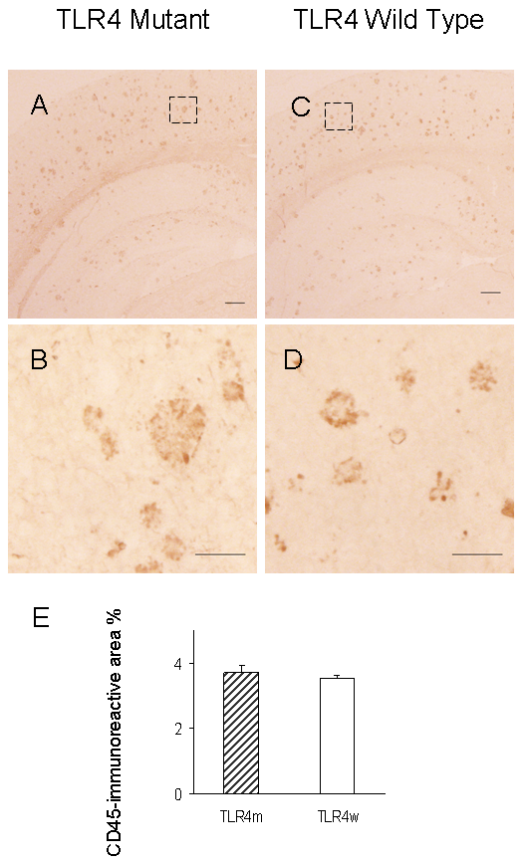
CD45-immunoreactive cells in TLR4m and TLR4w AD mice. The frozen sections of cerebral cortices from TLR4m (A and B) and TLR4w (C and D) AD mice were stained with anti-CD45 antibody. Scale bars are 50 μm in A and C, and 10 μm in B and D. The middle images (B and D) are a high magnification of the areas indicated by the squares in the top images (A and C). CD45 immunoreactive area percentages of TLR4m and TLR4w AD mice were shown as a bar graph (means ± SE) (E). Approximately 15 fields of the cerebral cortex (0.5 to 1 mm^2 ^each, using a 10× objective and 1× eyepiece lens) from 4 – 5 coronal brain sections, each separated by more than 240 μm interval, from each mouse were analyzed. TLR4m and TLR4w represent TLR4m and TLR4w AD mice, respectively.

**Figure 4:**
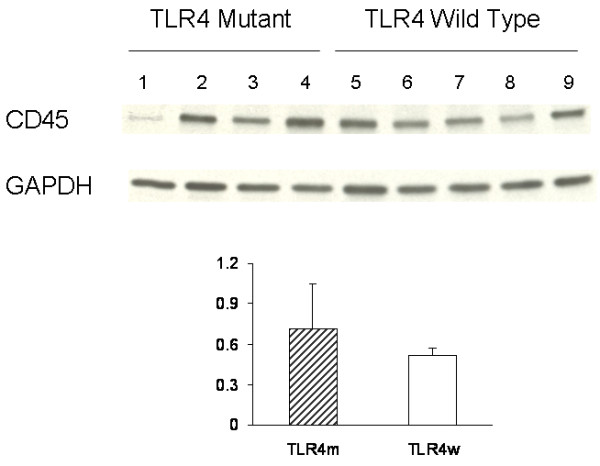
Levels of CD45 and GAPDH in the cerebral tissue lysates were determined by immunoblotting using anti-CD45 and anti-GAPDH antibodies, respectively. The bar graph represents densitometric quantification of CD45 after normalization with GAPDH (means ± SE). The mean of CD45 levels in TLR4m AD mice is greater than that in TLR4w AD mice but not statistically significant. Lane 1 through 4 are tissue lysates from TLR4m AD mice and lane 5 through 9 are from TLR4w AD mice.

The common leukocyte antigen, CD45, was used to assess the invasion of the brain by blood-derived leukocytes. The cerebral cortex of TLR4m and TLR4w AD mice was immunostained with anti-CD45 antibody (Fig. 3 A through D). No significant difference was observed between the two groups: 3.74 ± 0.19 and 3.54 ± 0.12% for TLR4m and TLR4w AD mice, respectively (P = 0.37) (Fig. 3E). CD45 immunoreactivity in the brains of TLR4m and TLR4w non-AD mice was unremarkable (data not shown). The common leukocyte antigen in the cerebral tissue lysates was further determined by immunoblot analysis (Fig. 4). There was no difference between TLR4m and TLR4w AD mice in expression levels of CD45 (Fig. 4).

### Reactive astrocytes

**Figure 5:**
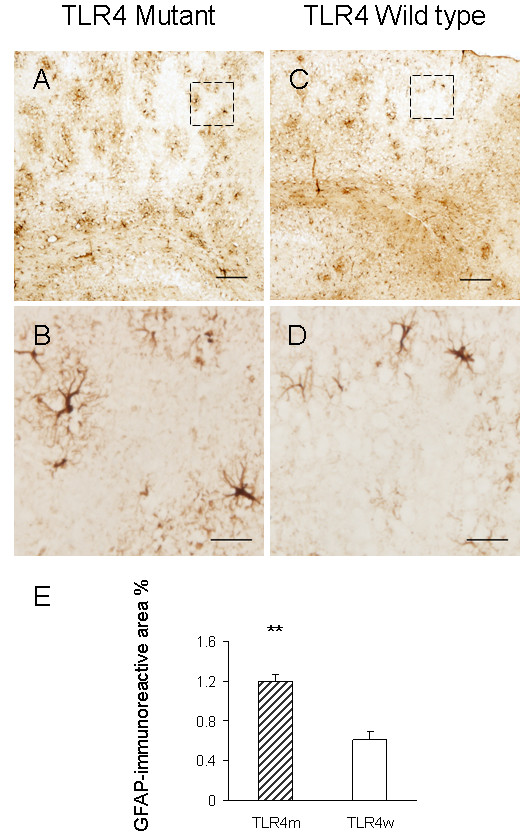
Reactive astrocytes in TLR4m AD mice increase compared to TLR4w AD mice by immunohistochemistry. The frozen sections of cerebral cortices from TLR4m (A and B) and TLR4w (C and D) AD mice were stained with anti-GFAP antibody. Scale bars are 50 μm in A and C, and 10 μm in B and D. The middle images (B and D) are a high magnification of the areas indicated by the squares in the top images (A and C). GFAP immunoreactive area percentages of TLR4m and TLR4w AD mice were shown as a bar graph (means ± SE, **P < 0.001) (E). Approximately 15 fields of the cerebral cortex (0.5 to 1 mm^2^each, using a 10× objective and 1× eyepiece lens) from 4 – 5 coronal brain sections, each separated by more than 240 μm interval, from each mouse were analyzed. TLR4m and TLR4w represent TLR4m and TLR4w AD mice, respectively.

**Figure 6:**
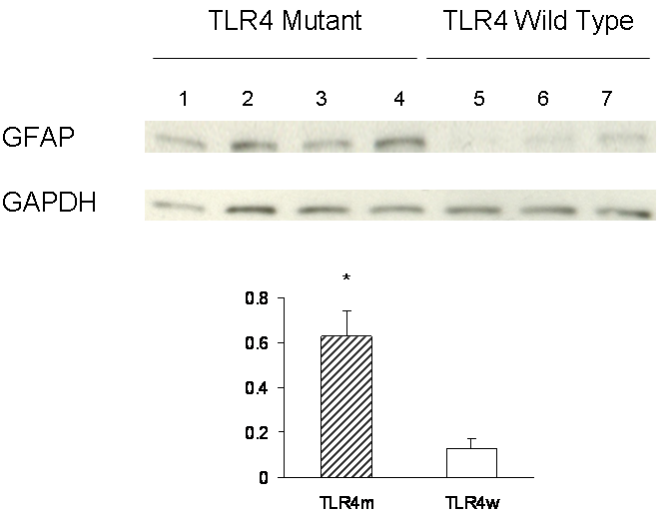
Levels of GFAP and GAPDH in the cerebral tissue lysates were determined by immunoblotting using anti-GFAP and anti-GAPDH antibodies, respectively. The bar graph represents densitometric quantification of GFAP after normalization with GAPDH (means ± SE). GFAP levels in TLR4m AD mice are greater than those in TLR4w AD mice (*P < 0.05). Lane 1 through 4 are tissue lysates from TLR4m AD mice and lane 5 through 7 are from TLR4w AD mice.

Reactive astrocytes in the brains of TLR4m and TLR4w AD mice were detected by immunohistochemistry using an antibody against an astrocyte marker, GFAP (Fig. 5A through 5D). Morphometric analysis indicated that the cerebral cortex of TLR4m AD mice (1.20 ± 0.07%) had a greater degree of astrocytosis compared with TLR4w AD mice (0.61 ± 0.08%, P = 0.0006) (Fig. 5E). Astrocyte immunoreactivity for GFAP in the cortex of non-AD mice was unremarkable (data not shown). Immunoblot analysis was also used to determine GFAP expression levels (Fig. 6). Densitometric scanning of the immunoblot confirmed that GFAP expression levels in the cerebral tissue lysates from TLR4m AD mice were higher than those from TLR4w AD mice (P = 0.016) (Fig. 6). Thus, astrocytosis in TLR4m AD mice was greater than that in TLR4w AD mice. These results are in agreement with our previous report that Aβ load in the cerebral cortex of TLR4m AD mice is greater than that of TLR4w AD mice [17].

### Levels of cytokines and chemokines

**Figure 7:**
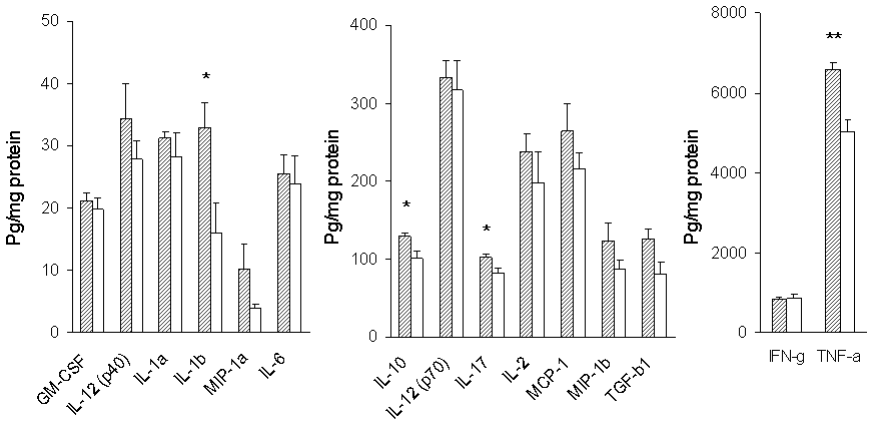
Levels of cerebral cytokines and chemokines in TLR4w AD and non-AD mice at the age of 13–15 months. Levels of cytokines and chemokines in the cerebral tissue lysates were determined by multiplex cytokine/chemokine array analysis. Hatched and open bars represent levels of cytokines and chemokines in TLR4w AD and non-AD mice, respectively. Mean concentrations ± SE are expressed in picograms per milligram of brain protein. *P < 0.05, **P = 0.001.

**Figure 8:**
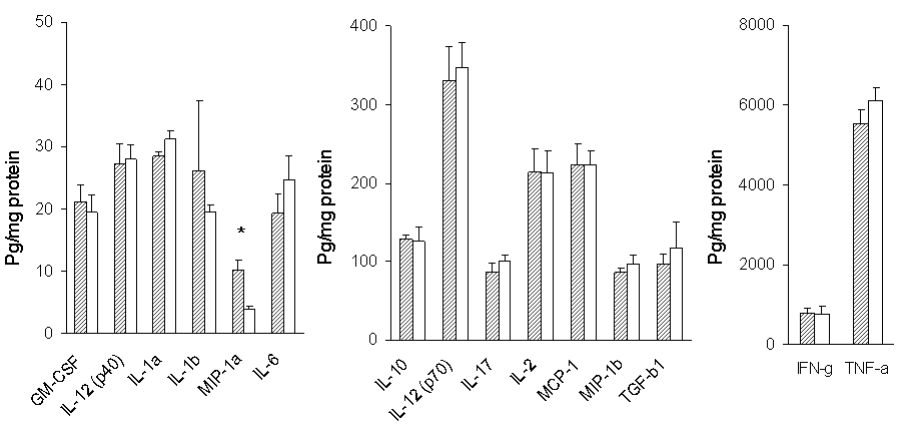
Levels of cerebral cytokines and chemokines in TLR4m AD and non-AD mice at the age of 13–15 months. Levels of cytokines and chemokines in the cerebral tissue lysates were determined by multiplex cytokine/chemokine array analysis. Hatched and open bars represent levels of cytokines and chemokines in TLR4m AD and non-AD mice, respectively. Mean concentrations ± SE are expressed in picograms per milligram of brain protein. *P < 0.05.

Levels of cytokines and chemokines in the cerebral extracts from the four experimental groups of mice were determined by multiplex cytokine/chemokine array analysis using the bead-based array technology [34]. Cytokines included IL-1α, IL-1β, IL-2, IL-3, IL-4, IL-6, IL-10, IL-12 (p40), IL-12(p70), IL-17, GM-CSF, IFN-γ, TGF-β1, and TNF-α. Chemokines determined were MCP-1, MIP-1α and MIP-1β. IL-3 and IL-4 in the cerebral tissue lysates were less than 2.5 and 4.3 pg/ml, respectively, which are the minimum detectable levels. Therefore, IL-3 and IL-4 were omitted from analysis. The levels of these cytokines and chemokines are presented as bar graphs in Fig. 7 and 8. Levels of IL-1β, IL-10, IL-17 and TNF-α increased in the cerebrum of TLR4w AD mice compared to TLR4w non-AD mice (P = 0.029, 0.013, 0.031 and 0.001, respectively) (Fig. 7). There were no significant differences in the other cytokines and chemokines between these TLR4w groups. Levels of MIP-1α increased in TLR4m AD mice compared to TLR4m non-AD mice (P = 0.006) (Fig. 8). There were no significant differences in any of the other cytokines and chemokines between these TLR4m groups. Levels of TNF-α and MIP-1β in TLR4w AD mice were higher than those in TLR4m AD mice (P = 0.014 and 0.019, respectively). There were no significant differences in the other cytokines and chemokines between these transgenic groups. Although levels of many cytokines such as IL-1, IL-2, IL-6, IL-10, IL-12, IL-17 and TGF-β1 in TLR4m non-AD mice on average are higher than those in TLR4w non-AD mice, the differences are not statistically significant. Levels of TNF-α in TLR4m non-AD mice, however, were higher than those in TLR4w non-AD mice (P = 0.048), suggesting compensatory upregulation of some cytokines in TLR4m mice.

## Discussion

Neuroinflammation characterized by increases in activated microglia and cytokines is thought to modulate the AD progression [1,2,35-37]. Such neuroinflammatory changes in AD are partly recapitulated in transgenic animal models of AD [9]. Here we demonstrated that levels of IL-1β, IL-10, IL-17 and TNF-α increased in the cerebrum of TLR4w AD mice compared to TLR4w non-AD mice. The levels of such cytokines in TLR4m AD mice were not different from those in TLR4m non-AD mice. These results indicate that TLR4 signaling mediates Aβ-induced upregulation of IL-1β, IL-10, IL-17 and TNF-α in TLR4w AD mice. It has been shown that fibrillar Aβ interacted with CD14 (TLR2 and TLR4 accessory protein) and was phagocytosed by cultured microglia and monocytes, resulting in activation of the cells as well as increased secretion of cytokines [38,39]. Aggregated Aβ failed to activate cultured microglia from TLR4m C3H/HeJ mice resulting in a diminished release of IL-6, TNF-α and nitric oxide compared to microglia from TLR4w C3H/HeN mice [40]. TLR4 neutralizing antibody also blocked Aβ-induced activation of human monocytes [40]. Upregulation of TLR4 and the association of CD14 with microglia in the brains of AD patients were demonstrated by immunohistochemistry [38,40]. We also found TLR4 upregulation in activated microglia which were intimately associated with Aβ deposits in Tg2576 mice (data not shown). These findings suggest that aggregated Aβ can induce activation of microglia and upregulation of certain cytokines through TLR4 signaling.

IL-1β, IL-10, IL-17 and TNF-α were upregulated in the brains of AD mice in a TLR4-dependent manner. As demonstrated by many investigators, expression of IL-1β and TNF-α is induced in cultured microglia and astrocytes in response to Aβ treatment [2,37]. Many investigators reported upregulation of IL-1β and TNF-α in AD mouse models and AD patients although some reports presented controversial data [2,9,37]. Upregulation of IL-10 was also found in tissue samples from AD mouse models and AD patients [41-43]. Because cytokines regulate the intensity and duration of immune responses [44] and because overexpression of IL-1β and TGF-β reduced Aβ load in the brains of AD mouse models, upregulation of some of these cytokines might have contributed to reducing Aβ load in TLR4w AD mice [17].

To the best of our knowledge, upregulation of IL-17 has not been reported in tissue samples from AD mouse models and AD patients. IL-17 is a proinflammatory cytokine produced from Th17 T cells that develop from a lineage distinct from the T helper-type 1 and 2 lineages [45]. IL-17 has been demonstrated to play important roles in the pathogenesis of autoimmune diseases such as multiple sclerosis and its animal models, experimental autoimmune encephalomyelitis [46-48]. Upon stimulation with IL-1β, IL-17 is produced by microglia, also [49]. Therefore, upregulation of IL-17 may be due to increased levels of IL-1β in the brains of TLR4w AD mice. The role of IL-17 in the pathogenesis of AD remains to be investigated.

Activated microglia and reactive astrocytes are closely associated with fibrilar Aβ deposits but are rarely found in immediate proximity to diffuse Aβ deposits [9]. TLR4m AD mice at 14–16 months of age showed an increase in fibrillar Aβ deposits compared to TLR4w mice [17]. Immunoreactivity for CD11b and GFAP, markers for activated microglia and astrocyte, respectively, increased in 13–15 month-old TLR4m AD mice compared to TLR4w AD mice. On the other hand, immunoreactivity for CD45 (migratory leukocytes) in TLR4m AD mice was not different from that in TLR4w AD mice. In CCR2-deficient or C3 inhibitor-overexpressing mice, however, an increase in Aβ levels in the brain was associated with a decrease in microglial cells [23,24]. Overexpression of TGF-β1 or IL-1β increased activated microglia and reduced Aβ load in the brains of AD mouse models [24,27]. Therefore, we expected less activated microglia and more Aβ load in TLR4m AD mice compared to TLR4w AD mice. The reasons for this discrepancy are not clear. This discrepancy, however, may be due to use of CD11b as a marker for activated microglia because microglia at distinct activation states differentially express several activation markers [9,19]. Alternatively, an acute state of activated microglia needs to be investigated to observe less activated microglia in TLR4m mice because AD mice develop amyloid deposits as early as 4–5 months of age [26,50]. Different aggregation states of Aβ react with multiple cell surface proteins/receptors other than CD14/TLR4 [51]. Such proteins/receptors may activate microglia in a chronic state without the beneficial effects. We are currently investigating activation of microglia and expression of cytokines in TLR4m AD mice at younger ages.

## Conclusion

In summary, we found upregulation of IL-1β, IL-10, IL-17 and TNF-α in TLR4w AD mice compared to non-AD mice. Such upregulation was not observed in TLR4m AD mice. Our results suggest that TLR4 signaling may be involved in the AD progression through upregulation of certain cytokines. Because IL-1β and TNF-α can modulate the AD-like pathology progression in AD mouse models, TLR4 signaling can be a new therapeutic target for AD.

## Abbreviations

AD: Alzheimer's disease; TLRs: toll-like receptors; TNF: tumor necrosis factor; IL: interleukin; Aβ: aggregated amyloid β-protein; NSAID: nonsteroidal anti-inflammatory drugs; APP: amyloid β-protein precursor; IFN: interferon; BACE1: β-site APP-cleaving enzyme; TLR2: toll-like receptor 2; LPS: lipopolysaccharide; MGAT3: mannosyl-glycoprotein 4-β-N-acetylglucosaminyltransferase; GFAP: glial fibrillary acidic protein; PVDF: polyvinylidine difluoride; PBS: phosphate buffered saline; GAPDH: glyceraldehyde-3-phosphate dehydrogenase; ANOVA: analysis of variance; TBS: Tris-buffered saline; BSA: bovine serum albumin; TBST: triton-X-100 TBS; GM-CSF: granulocyte macrophage colony-stimulating factor; TGF: transforming growth factor; MCP-1: monocyte chemotactic protein-1; MIP: macrophage inflammatory protein

## Competing interests

The authors declare that they have no competing interests.

## Authors' contributions

KF designed the study and reviewed the data, KF and JJ wrote the manuscript, JJ and HDK performed immunohistochemistry and protein assays, JJ performed western blotting, morphometric and statistical analyses, JAM performed production and genotyping of experimental animals, LL provided experimental animals and consultation and reviewed the data.

## References

[B1] Akiyama H, Barger S, Barnum S, Bradt B, Bauer J, Cole GM, Cooper NR, Eikelenboom P, Emmerling M, Fiebich BL, Finch CE, Frautschy S, Griffin WS, Hampel H, Hull M, Landreth G, Lue L, Mrak R, Mackenzie IR, McGeer PL, O'Banion MK, Pachter J, Pasinetti G, Plata-Salaman C, Rogers J, Rydel R, Shen Y, Streit W, Strohmeyer R, Tooyoma I, Van Muiswinkel FL, Veerhuis R, Walker D, Webster S, Wegrzyniak B, Wenk G, Wyss-Coray T (2000). Inflammation and Alzheimer's disease. Neurobiol Aging.

[B2] Heneka MT, O'Banion MK (2007). Inflammatory processes in Alzheimer's disease. J Neuroimmunol.

[B3] Breitner JC, Welsh KA, Helms MJ, Gaskell PC, Gau BA, Roses AD, Pericak-Vance MA, Saunders AM (1995). Delayed onset of Alzheimer's disease with nonsteroidal anti-inflammatory and histamine H2 blocking drugs. Neurobiol Aging.

[B4] Breitner JC, Gau BA, Welsh KA, Plassman BL, McDonald WM, Helms MJ, Anthony JC (1994). Inverse association of anti-inflammatory treatments and Alzheimer's disease: initial results of a co-twin control study. Neurology.

[B5] McGeer PL, McGeer E, Rogers J, Sibley J (1990). Anti-inflammatory drugs and Alzheimer disease. Lancet.

[B6] Stewart WF, Kawas C, Corrada M, Metter EJ (1997). Risk of Alzheimer's disease and duration of NSAID use. Neurology.

[B7] Szekely CA, Thorne JE, Zandi PP, Ek M, Messias E, Breitner JC, Goodman SN (2004). Nonsteroidal anti-inflammatory drugs for the prevention of Alzheimer's disease: a systematic review. Neuroepidemiology.

[B8] Veld BA, Ruitenberg A, Launer LJ (2000). Duration of nonsteroidal antiinflammatory drug use and risk of Alzheimer's disease.  The Rotterdam study. Neurobiol Aging.

[B9] Morgan D, Gordon MN, Tan J, Wilcock D, Rojiani AM (2005). Dynamic complexity of the microglial activation response in transgenic models of amyloid deposition: implications for Alzheimer therapeutics. J Neuropathol Exp Neurol.

[B10] Lim GP, Yang F, Chu T, Chen P, Beech W, Teter B, Tran T, Ubeda O, Ashe KH, Frautschy SA, Cole GM (2000). Ibuprofen suppresses plaque pathology and inflammation in a mouse model for Alzheimer's disease. J Neurosci.

[B11] Lim GP, Yang F, Chu T, Gahtan E, Ubeda O, Beech W, Overmier JB, Hsiao-Ashec K, Frautschy SA, Cole GM (2001). Ibuprofen effects on Alzheimer pathology and open field activity in APPsw transgenic mice. Neurobiol Aging.

[B12] He P, Zhong Z, Lindholm K, Berning L, Lee W, Lemere C, Staufenbiel M, Li R, Shen Y (2007). Deletion of tumor necrosis factor death receptor inhibits amyloid beta generation and prevents learning and memory deficits in Alzheimer's mice. J Cell Biol.

[B13] Yamamoto M, Kiyota T, Horiba M, Buescher JL, Walsh SM, Gendelman HE, Ikezu T (2007). Interferon-gamma and tumor necrosis factor-alpha regulate amyloid-beta plaque deposition and beta-secretase expression in Swedish mutant APP transgenic mice. Am J Pathol.

[B14] Chen K, Iribarren P, Hu J, Chen J, Gong W, Cho EH, Lockett S, Dunlop NM, Wang JM (2006). Activation of toll-like receptor 2 on microglia promotes cell up-take of Alzheimer's disease-associated amyloid beta peptide. J Biol Chem.

[B15] Iribarren P, Chen K, Hu J, Gong W, Cho EH, Lockett S, Uranchimeg B, Wang JM (2005). CpG-containing oligodeoxynucleotide promotes microglial cell uptake of amyloid beta 1-42 peptide by up-regulating the expression of the G-protein- coupled receptor mFPR2. FASEB J.

[B16] Kakimura J, Kitamura Y, Takata K, Umeki M, Suzuki S, Shibagaki K, Taniguchi T, Nomura Y, Gebicke-Haerter PJ, Smith MA, Perry G, Shimohama S (2002). Microglial activation and amyloid-beta clearance induced by exogenous heat-shock proteins. FASEB J.

[B17] Tahara K, Kim HD, Jin J, Maxwell JA, Li L, Fukuchi KI (2006). Role of toll-like receptor signalling in A{beta} uptake and clearance. Brain.

[B18] DiCarlo G, Wilcock D, Henderson D, Gordon M, Morgan D (2001). Intrahippocampal LPS injections reduce Abeta load in APP+PS1 transgenic mice. Neurobiol Aging.

[B19] Herber DL, Mercer M, Roth LM, Symmonds K, Maloney J, Wilson N, Freeman MJ, Morgan D, Gordon MN (2007). Microglial activation is required for Abeta clearance after intracranial injection of lipopolysaccharide in APP transgenic mice. J Neuroimmune Pharmacol.

[B20] Simard AR, Soulet D, Gowing G, Julien JP, Rivest S (2006). Bone marrow-derived microglia play a critical role in restricting senile plaque formation in Alzheimer's disease. Neuron.

[B21] Malm TM, Koistinaho M, Parepalo M, Vatanen T, Ooka A, Karlsson S, Koistinaho J (2005). Bone-marrow-derived cells contribute to the recruitment of microglial cells in response to beta-amyloid deposition in APP/PS1 double transgenic Alzheimer mice. Neurobiol Dis.

[B22] Fiala M, Liu PT, Espinosa-Jeffrey A, Rosenthal MJ, Bernard G, Ringman JM, Sayre J, Zhang L, Zaghi J, Dejbakhsh S, Chiang B, Hui J, Mahanian M, Baghaee A, Hong P, Cashman J (2007). Innate immunity and transcription of MGAT-III and Toll-like receptors in Alzheimer's disease patients are improved by bisdemethoxycurcumin. Proc Natl Acad Sci U S A.

[B23] El Khoury J, Toft M, Hickman SE, Means TK, Terada K, Geula C, Luster AD (2007). Ccr2 deficiency impairs microglial accumulation and accelerates progression of Alzheimer-like disease. Nat Med.

[B24] Wyss-Coray T, Yan F, Lin AH, Lambris JD, Alexander JJ, Quigg RJ, Masliah E (2002). Prominent neurodegeneration and increased plaque formation in complement-inhibited Alzheimer's mice. Proc Natl Acad Sci U S A.

[B25] Wyss-Coray T, Lin C, Yan F, Yu GQ, Rohde M, McConlogue L, Masliah E, Mucke L (2001). TGF-beta1 promotes microglial amyloid-beta clearance and reduces plaque burden in transgenic mice. Nat Med.

[B26] Jankowsky JL, Fadale DJ, Anderson J, Xu GM, Gonzales V, Jenkins NA, Copeland NG, Lee MK, Younkin LH, Wagner SL, Younkin SG, Borchelt DR (2004). Mutant presenilins specifically elevate the levels of the 42 residue beta-amyloid peptide in vivo: evidence for augmentation of a 42-specific gamma secretase. Hum Mol Genet.

[B27] Shaftel SS, Kyrkanides S, Olschowka JA, Miller JN, Johnson RE, O'Banion MK (2007). Sustained hippocampal IL-1 beta overexpression mediates chronic neuroinflammation and ameliorates Alzheimer plaque pathology. J Clin Invest.

[B28] Gordon S (2002). Pattern recognition receptors: doubling up for the innate immune response. Cell.

[B29] Akira S, Uematsu S, Takeuchi O (2006). Pathogen recognition and innate immunity. Cell.

[B30] Doyle SE, O'Connell RM, Miranda GA, Vaidya SA, Chow EK, Liu PT, Suzuki S, Suzuki N, Modlin RL, Yeh WC, Lane TF, Cheng G (2004). Toll-like receptors induce a phagocytic gene program through p38. J Exp Med.

[B31] McKimmie CS, Roy D, Forster T, Fazakerley JK (2006). Innate immune response gene expression profiles of N9 microglia are pathogen-type specific. J Neuroimmunol.

[B32] Bsibsi M, Ravid R, Gveric D, van Noort JM (2002). Broad expression of Toll-like receptors in the human central nervous system. J Neuropathol Exp Neurol.

[B33] Olson JK, Miller SD (2004). Microglia initiate central nervous system innate and adaptive immune responses through multiple TLRs. J Immunol.

[B34] Opalka D, Lachman CE, MacMullen SA, Jansen KU, Smith JF, Chirmule N, Esser MT (2003). Simultaneous quantitation of antibodies to neutralizing epitopes on virus-like particles for human papillomavirus types 6, 11, 16, and 18 by a multiplexed luminex assay. Clin Diagn Lab Immunol.

[B35] Wyss-Coray T (2006). Inflammation in Alzheimer disease: driving force, bystander or beneficial response?. Nat Med.

[B36] Eikelenboom P, Veerhuis R, Scheper W, Rozemuller AJ, van Gool WA, Hoozemans JJ (2006). The significance of neuroinflammation in understanding Alzheimer's disease. J Neural Transm.

[B37] Rojo LE, Fernandez JA, Maccioni AA, Jimenez JM, Maccioni RB (2008). Neuroinflammation: implications for the pathogenesis and molecular diagnosis of Alzheimer's disease. Arch Med Res.

[B38] Liu Y, Walter S, Stagi M, Cherny D, Letiembre M, Schulz-Schaeffer W, Heine H, Penke B, Neumann H, Fassbender K (2005). LPS receptor (CD14): a receptor for phagocytosis of Alzheimer's amyloid peptide. Brain.

[B39] Udan ML, Ajit D, Crouse NR, Nichols MR (2008). Toll-like receptors 2 and 4 mediate Abeta(1-42) activation of the innate immune response in a human monocytic cell line. J Neurochem.

[B40] Walter S, Letiembre M, Liu Y, Heine H, Penke B, Hao W, Bode B, Manietta N, Walter J, Schulz-Schuffer W, Fassbender K (2007). Role of the toll-like receptor 4 in neuroinflammation in Alzheimer's disease. Cell Physiol Biochem.

[B41] Benzing WC, Wujek JR, Ward EK, Shaffer D, Ashe KH, Younkin SG, Brunden KR (1999). Evidence for glial-mediated inflammation in aged APP(SW) transgenic mice. Neurobiol Aging.

[B42] Apelt J, Schliebs R (2001). Beta-amyloid-induced glial expression of both pro- and anti-inflammatory cytokines in cerebral cortex of aged transgenic Tg2576 mice with Alzheimer plaque pathology. Brain Res.

[B43] Lombardi VR, Garcia M, Rey L, Cacabelos R (1999). Characterization of cytokine production, screening of lymphocyte subset patterns and in vitro apoptosis in healthy and Alzheimer's Disease (AD) individuals. J Neuroimmunol.

[B44] Tuppo EE, Arias HR (2005). The role of inflammation in Alzheimer's disease. Int J Biochem Cell Biol.

[B45] Harrington LE, Hatton RD, Mangan PR, Turner H, Murphy TL, Murphy KM, Weaver CT (2005). Interleukin 17-producing CD4+ effector T cells develop via a lineage distinct from the T helper type 1 and 2 lineages. Nat Immunol.

[B46] Hofstetter HH, Ibrahim SM, Koczan D, Kruse N, Weishaupt A, Toyka KV, Gold R (2005). Therapeutic efficacy of IL-17 neutralization in murine experimental autoimmune encephalomyelitis. Cell Immunol.

[B47] Ishizu T, Osoegawa M, Mei FJ, Kikuchi H, Tanaka M, Takakura Y, Minohara M, Murai H, Mihara F, Taniwaki T, Kira J (2005). Intrathecal activation of the IL-17/IL-8 axis in opticospinal multiple sclerosis. Brain.

[B48] Sutton C, Brereton C, Keogh B, Mills KH, Lavelle EC (2006). A crucial role for interleukin (IL)-1 in the induction of IL-17-producing T cells that mediate autoimmune encephalomyelitis. J Exp Med.

[B49] Kawanokuchi J, Shimizu K, Nitta A, Yamada K, Mizuno T, Takeuchi H, Suzumura A (2007). Production and functions of IL-17 in microglia. J Neuroimmunol.

[B50] Jankowsky JL, Slunt HH, Ratovitski T, Jenkins NA, Copeland NG, Borchelt DR (2001). Co-expression of multiple transgenes in mouse CNS: a comparison of strategies. Biomol Eng.

[B51] Verdier Y, Zarandi M, Penke B (2004). Amyloid beta-peptide interactions with neuronal and glial cell plasma membrane: binding sites and implications for Alzheimer's disease. J Pept Sci.

